# Clinical Relevance of Senior‐Supervised Transthoracic Echocardiography in Clinical Practice and Research: An Editorial Commentary and Systematic Review

**DOI:** 10.1111/echo.70085

**Published:** 2025-01-24

**Authors:** Daniel A. Morris

**Affiliations:** ^1^ Deutsches Herzzentrum der Charité, Department of Cardiology Angiology and Intensive Care Medicine Berlin Germany

1

In clinical practice, senior cardiologists in echocardiography laboratories frequently find clinically significant findings during supervised transthoracic echocardiography (TTE) that less experienced colleagues had not identified [1]. These findings, which are exclusively uncovered during senior‐supervised TTE, encompass a wide range of abnormalities, from apical left ventricular (LV) thrombus to subtle changes in LV wall thickness [1]. However, the most commonly affected parameters or evaluations are the LV ejection fraction (LVEF) and the severity of valvular heart disease [1, 2, 3].

Several studies have highlighted the clinical importance of senior‐supervised TTE in determining the final value of LVEF that should be reported [2]. In effect, as demonstrated in the present systematic review (see Table 1), the absolute mean difference in LVEF between evaluations conducted by senior cardiologists and those by non‐senior practitioners is approximately 3% absolute LVEF units, with a range of 0%–8% (95% confidence interval: 1.8%–4.4%) (see Table 1). These discrepancies have significant clinical implications. For instance, a senior‐supervised evaluation might classify a patient's LVEF as < 35% rather than > 40% (see Table 1). This change in LVEF value would indicate the initiation of specific heart failure therapies or consideration of advanced interventions such as cardiac resynchronization therapy or implantable cardioverter–defibrillators, decisions that might not have been made without senior supervision.

**TABLE 1:** Systematic review on absolute mean difference in 2D LVEF between senior cardiologists and non‐senior practitioners.

Study	Sample size	Absolute mean difference in LVEF (LVEF units—bias)	Rate of inaccurate determination of LVEF < 50% by non‐senior	Rate of inaccurate determination of LVEF < 40% by non‐senior	Rate of inaccurate determination of LVEF < 30% by non‐senior
Moore et al. 2002 [4]	51	2.0 units (overestimation)	23%	50%	20%
Hole et al. 2002* [5]	756	0.6 unit (underestimation)	n/a	n/a	n/a
Randazzo et al. 2003 [6]	115	1.2 unit (underestimation)	7.7%	52.2%	29.6%
Dart et al. 2007* [7]	7,016	8.0 units (overestimation)	n/a	7.1%	n/a
Culp et al. 2010 [8]	40	5.9 units (underestimation)	n/a	n/a	n/a
Bull et al. 2011 [9]	64	3.5 units (overestimation)	n/a	n/a	n/a
Bustam et al. 2014 [10]	100	2.1 units (overestimation)	6.0%	n/a	5.9%
Medvedofsky et al. 2017 [11]	30	3.5 units (underestimation)	n/a	n/a	n/a
Franchi et al. 2018 [12]	29	0.3 unit (underestimation)	n/a	n/a	n/a
Karlsen et al. 2019 [13]	47	4.08 units (underestimation)	n/a	6.4%	n/a
Baldea et al. 2021 [14]	54	5.1 units (overestimation)	n/a	n/a	n/a
Varudo et al. 2022 [15]	95	0 unit	n/a	n/a	n/a
He et al. 2023 [16]	1,755	3.77 units (overestimation)	n/a	n/a	n/a
Akil et al. 2025 [17]	140	3.2 units (overestimation)	n/a	n/a	n/a
**Total number of studies**: 14	**Total sample size**: 10,292	**Average/pooled absolute mean difference in LVEF (LVEF units—bias)**: 3.0 units (range 0–8 units; 95% CI 1.8–4.4 units)	**Average rate of inaccurate determination of LVEF < 50% by non‐senior** 12.2%	**Average rate of inaccurate determination of LVEF < 40% by non‐senior**: 28.9%	**Average rate of inaccurate determination of LVEF < 30% by non‐senior**: 18.5%

*Note*: The systematic review was based on a PubMed search for studies in adults published in the English language up to January 12, 2025, using the following search terms: LVEF AND expert; LVEF AND reproducibility; LVEF AND experienced. The systematic review aimed to compare two‐dimensional (2D) left ventricular ejection fraction (LVEF) between senior cardiologists and non‐senior practitioners. The main inclusion criteria and primary analysis for the systematic review was the mean absolute difference in LVEF (LVEF units) between senior (or expert) versus non‐senior (or non‐expert) medical practitioners. LVEF, left ventricular ejection fraction. CI, confidence interval. * indicates a comparison of LVEF analysis between senior cardiologists at an echocardiography core laboratory and medical practitioners at study centers.

The evaluation of valvular heart disease severity is another critical area where senior‐supervised TTE plays a pivotal role [3]. It is not uncommon for senior‐supervised TTE to identify severe aortic stenosis (AS), severe mitral regurgitation, or severe tricuspid regurgitation, whereas less experienced colleagues reported only moderate severity [1, 3]. In cases of AS, discrepancies often arise from differences in the measurement of the LV outflow tract (LVOT) diameter and the peak aortic valve velocity obtained via continuous‐wave Doppler [3, 18]. In this issue of the journal, Velders et al. demonstrated significant and clinically relevant differences in LVOT diameter measurements between evaluations conducted by senior cardiologists in an academic echocardiography core laboratory and routine or screening measurements from study centers [19]. Similar findings have been corroborated by other studies comparing LVOT diameter and peak aortic valve velocity measurements in patients with AS and bioprosthetic aortic valves between academic echocardiography core laboratories or senior cardiologists and study centers [20, 21].

Based on the above‐mentioned evidence, the evidence shown in the current systematic review and current expert consensus recommendations [1, 2, 3, 4, 5, 6, 7, 8, 9, 10, 11, 12, 13, 14, 15, 16, 17, 18, 19, 20, 21, 22, 23, 24, 25, 26], senior supervision of TTE is clinically relevant in both clinical practice and research. Senior oversight ensures the accuracy of measurements and interpretations, minimizing the risk of critical errors in key parameters or evaluations such as LVEF and valvular disease severity [1–3, 19, 22–26]. Consequently, TTE assessments conducted in clinical or research echocardiography laboratories or in academic core laboratories should be supervised by experienced cardiologists [1–3, 19, 22–26]. Such practice is essential for preventing clinically significant mismeasurements or misinterpretations [1–3, 19, 22–26], thereby ensuring optimal patient care and the accuracy of research findings [1–3, 19, 22–26].

## Data Availability

n/a.
